# Prospective Evaluation of HIV Testing Technologies in a Clinical Setting: Protocol for Project DETECT

**DOI:** 10.2196/16332

**Published:** 2020-01-27

**Authors:** Joanne D Stekler, Lauren R Violette, Hollie A Clark, Sarah J McDougal, Lisa A Niemann, David A Katz, Pollyanna R Chavez, Laura G Wesolowski, Steven F Ethridge, Vanessa M McMahan, Andy Cornelius-Hudson, Kevin P Delaney

**Affiliations:** 1 Department of Medicine University of Washington Seattle, WA United States; 2 Department of Epidemiology University of Washington Seattle, WA United States; 3 Department of Global Health University of Washington Seattle, WA United States; 4 Division of HIV/AIDS Prevention Centers for Disease Control and Prevention Atlanta, GA United States; 5 HIV/STD Program Public Health–Seattle and King County Seattle, WA United States

**Keywords:** HIV testing, point-of-care tests, acute HIV infection, nucleic acid tests

## Abstract

**Background:**

HIV testing guidelines provided by the Centers for Disease Control and Prevention (CDC) are continually changing to reflect advancements in new testing technology. Evaluation of existing and new point-of-care (POC) HIV tests is crucial to inform testing guidelines and provide information to clinicians and other HIV test providers. Characterizing the performance of POC HIV tests using unprocessed specimens can provide estimates for the window period of detection, or the time from HIV acquisition to test positivity, which allows clinicians and other HIV providers to select the appropriate POC HIV tests for persons who may be recently infected with HIV.

**Objective:**

This paper describes the protocols and procedures used to evaluate the performance of the newest POC tests and determine their sensitivity during early HIV infection.

**Methods:**

Project DETECT is a CDC-funded study that is evaluating POC HIV test performance. Part 1 is a cross-sectional, retrospective study comparing behavioral characteristics and HIV prevalence of the overall population of the Public Health–Seattle & King County (PHSKC) Sexually Transmitted Disease (STD) Clinic to Project DETECT participants enrolled in part 2. Part 2 is a cross-sectional, prospective study evaluating POC HIV tests in real time using unprocessed whole blood and oral fluid specimens. A POC nucleic acid test (NAT) was added to the panel of HIV tests in June 2018. Part 3 is a longitudinal, prospective study evaluating seroconversion sensitivity of POC HIV tests through serial follow-up testing. For comparison, HIV-1 RNA and HIV-1/HIV-2 antigen/antibody tests are also performed for participants enrolled in part 2 or 3. A behavioral survey that collects information about demographics, history of HIV testing, STD history, symptoms of acute HIV infection, substance use, sexual behaviors in the aggregate and with recent partners, and use of pre-exposure prophylaxis and antiretroviral therapy is completed at each part 2 or 3 visit.

**Results:**

Between September 2015 and March 2019, there were 14,990 Project DETECT–eligible visits (part 1) to the PHSKC STD Clinic resulting in 1819 part 2 Project DETECT study visits. The longitudinal study within Project DETECT (part 3) enrolled 27 participants with discordant POC test results from their part 2 visit, and 10 (37%) were followed until they had fully seroconverted with concordant positive POC test results. Behavioral survey data and HIV test results, sensitivity, and specificity will be presented elsewhere.

**Conclusions:**

Studies such as Project DETECT are critical for evaluating POC HIV test devices as well as describing characteristics of persons at risk for HIV acquisition in the United States. HIV tests in development, including POC NATs, will provide new opportunities for HIV testing programs.

**International Registered Report Identifier (IRRID):**

RR1-10.2196/16332

## Introduction

The Centers for Disease Control and Prevention (CDC) provides guidelines for HIV testing in the United States and must continually update its guidance to reflect advancements in testing technology, availability of new tests, and test performance across various specimen types and during both early and established HIV infection [1]. Acute HIV infection, the period between first detection of viral markers of HIV infection and the development of a mature antibody response, is a period characterized by a high viral load and potential for false-negative HIV antibody tests, leaving individuals unaware of their HIV infection. These conditions lead to an elevated risk of HIV transmission to others during this earliest period of infection [2]. Due to the higher transmission risk during early infection, the CDC and Association of Public Health Laboratories published a new algorithm in 2014 for laboratory testing to help identify persons recently infected with HIV that incorporated the use of an HIV antigen/antibody (Ag/Ab) test, which can detect HIV sooner than tests that detect only antibodies [3-8].

Data released by the CDC in 2017 [7] showed that, for laboratory-based Ag/Ab testing, the median time from the estimated dates of HIV acquisition to test positivity (the window period) was 18 days (interquartile range [IQR] 13-24 days), and it was 44 days before all specimens tested positive. CDC, therefore, recommends that persons tested less than 45 days after being exposed to HIV who receive a negative result on a laboratory-based HIV Ag/Ab test should have a follow-up test at 45 days postexposure [9]. Similar estimates for window periods of point-of-care (POC) tests have been difficult to calculate because POC tests are intended for use with specimens that are difficult to store or not commercially available, including unprocessed blood and oral fluid. Thus, to date the CDC has not been able to update recommendations for when to retest after possible HIV exposure when using POC HIV tests.

In 2014, CDC and University of Washington (UW) began the Diagnostic Evaluation To Expand Critical Testing Technologies (Project DETECT). The goals of this project are to evaluate the (1) performance of the newest POC HIV tests with unprocessed whole blood and oral fluid specimens and (2) sensitivity of various POC HIV tests during early infection. Results from Project DETECT will be used to inform HIV testing guidelines and technical guidance and provide information to clinicians and other HIV test providers on the appropriate use of different POC HIV tests and retesting procedures. In this manuscript, we describe the protocol and study populations on which these test evaluations are based and the types of behavioral and laboratory data and specimens collected by the study.

## Methods

### Description of the Protocol for Project DETECT

#### Study Populations

**Figure 1:**
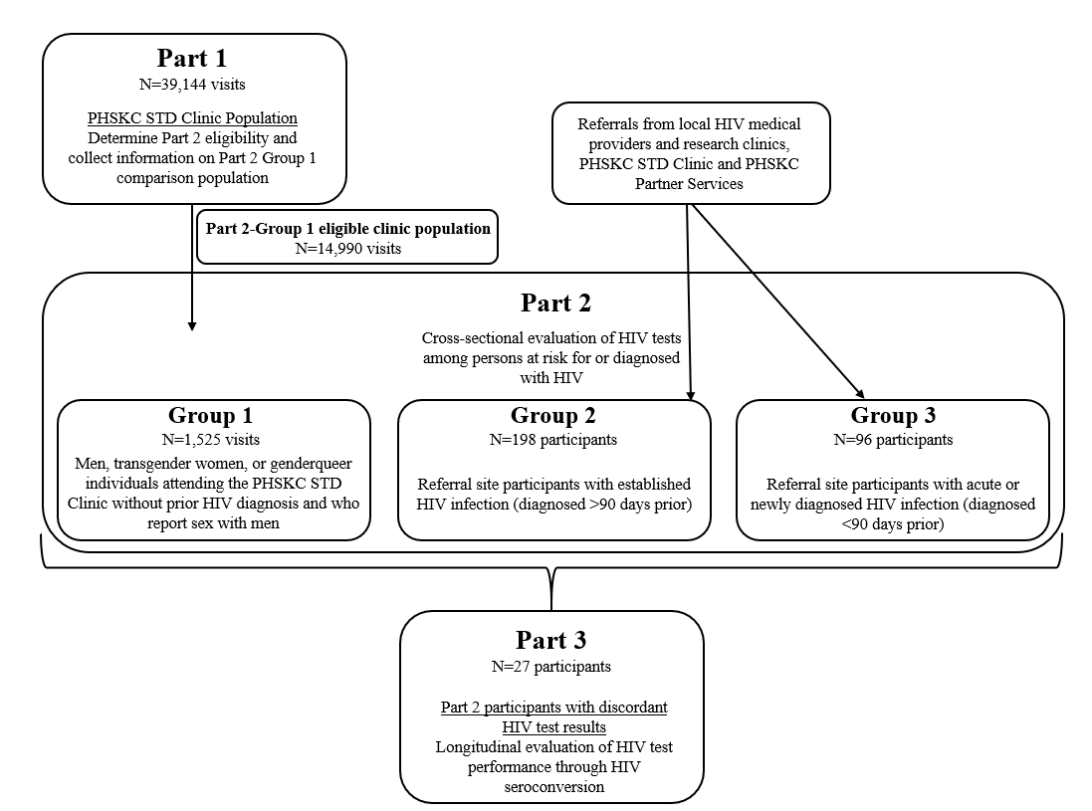
Project DETECT study design.

Project DETECT comprises three parts as depicted in Figure 1. It includes evaluations of POC HIV test performance in a cross section of participants with and without prior HIV diagnosis (part 2) and over the course of seroconversion (part 3) as well as a comparison of study participants to the overall clinic population from which study participants were drawn (part 1). The study received ethical approval from the UW Human Subjects Division (STUDY#00001637).

#### Project DETECT: Part 1

Project DETECT part 1 is a cross-sectional, retrospective study designed to use behavioral and testing data collected for clinical purposes to describe the population of the Public Health–Seattle & King County (PHSKC) Sexually Transmitted Diseases (STD) Clinic and compare behavioral characteristics and HIV prevalence of the overall clinic population with participants enrolled in part 2, described below. Persons are eligible for inclusion in part 1 if they are aged 14 years or older and presenting for a visit at the PHSKC STD Clinic. The majority of clinic clients are not eligible for part 2 based on criteria described in the next section. These participants receive standard care for sexual health services. A waiver of informed consent was granted by the UW Human Subjects Division for part 1 procedures.

#### Project DETECT: Part 2

Project DETECT part 2 is a cross-sectional, prospective study designed to evaluate the performance of new POC HIV tests in real time with unprocessed whole blood and oral fluid specimens. Part 2 participants are aged 18 years and older and are assigned to one of three groups:

- Part 2–group 1 consists of English-speaking cisgender men, transgender men, transgender women, and genderqueer individuals who have sex with men who come to the PHSKC STD Clinic seeking sexual health services and report being HIV negative or of unknown HIV status. Part 2–group 1 participants can reenroll as a part 2–group 1 participant every 90 days if they have concordant negative HIV POC and lab-based test results at their previous study visit and remain HIV negative or of unknown status.
- Part 2–group 2 consists of English-speaking HIV-positive persons whose first positive HIV test was more than 90 days prior to referral. Participants could be antiretroviral (ARV)-naïve, currently receiving antiretroviral therapy (ART), or persons who have discontinued ART.
- Part 2–group 3 consists of English- and Spanish-speaking persons with possible or diagnosed acute or early HIV infection. This group was included to enrich the sample of persons likely to have discordant HIV test results for part 3, as was done in a prior project [10]. All persons whose first positive HIV test was within 90 days preceding their part 2 visit, regardless of whether they were ARV-naïve or were newly taking ART, were enrolled into part 2–group 3.

The primary study site for part 2–group 1 recruitment and study-related activities is the PHSKC STD Clinic, drawing from a patient population of approximately 6500 patients and more than 10,000 visits per year.. Other referral and performance sites for part 2–groups 2 and 3 include the UW AIDS Clinical Trial Unit (ACTU), which has co-enrolled a subset of Project DETECT participants in an acute HIV infection treatment study, and other clinical providers or medical facilities in Western Washington. Project DETECT staff also screen daily patient intake lists for the HIV clinic that is co-located with the UW ACTU and approach patients for participation in part 2–group 2 or 3, as appropriate. All subjects provide verbal (part 2–groups 1 and 2) or written (part 2–group 3) informed consent for general study procedures as well as an additional consent specifically for specimen storage in a CDC repository. Participants are compensated $40 for their time; since October 2017, part 2–group 3 participants have received an additional $10 for fingerstick procedures.

#### Project DETECT: Part 3

Project DETECT part 3 is a prospective, longitudinal study designed to evaluate the seroconversion sensitivity of new POC HIV tests through serial follow-up and gather information on characteristics of persons undergoing seroconversion. Data from part 3 will be used to describe differences in the window periods of HIV tests by test and by specimen type.

Part 3 consists of English- or Spanish-speaking part 2 participants with discordant HIV test results (ie, at least one positive result and one or more negative results) who consent to longitudinal follow-up. Initially, any part 2 participant with discordant results was offered enrollment in part 3. Since April 2016, we have no longer offered part 3 enrollment to part 2–group 2 participants with negative POC test results, as discordant results in these participants likely indicates seroreversion.

Part 3 participants provide written informed consent for general study procedures and separate informed consent specifically for specimen storage in the CDC repository. Visits are targeted to be scheduled 3, 7, 10, 14, 21, 28, 42, 56, and 70 days after the part 2 visit and then monthly (Multimedia Appendix 1). Follow-up continues on schedule until participants test concordant positive on all POC HIV tests, concordant negative on all tests at two consecutive visits (indicating false-positive part 2 test results), or they complete one year of follow-up. Part 3 participants receive $50 at each study visit.

### Study Procedures

#### HIV Testing Procedures

**Figure 2:**
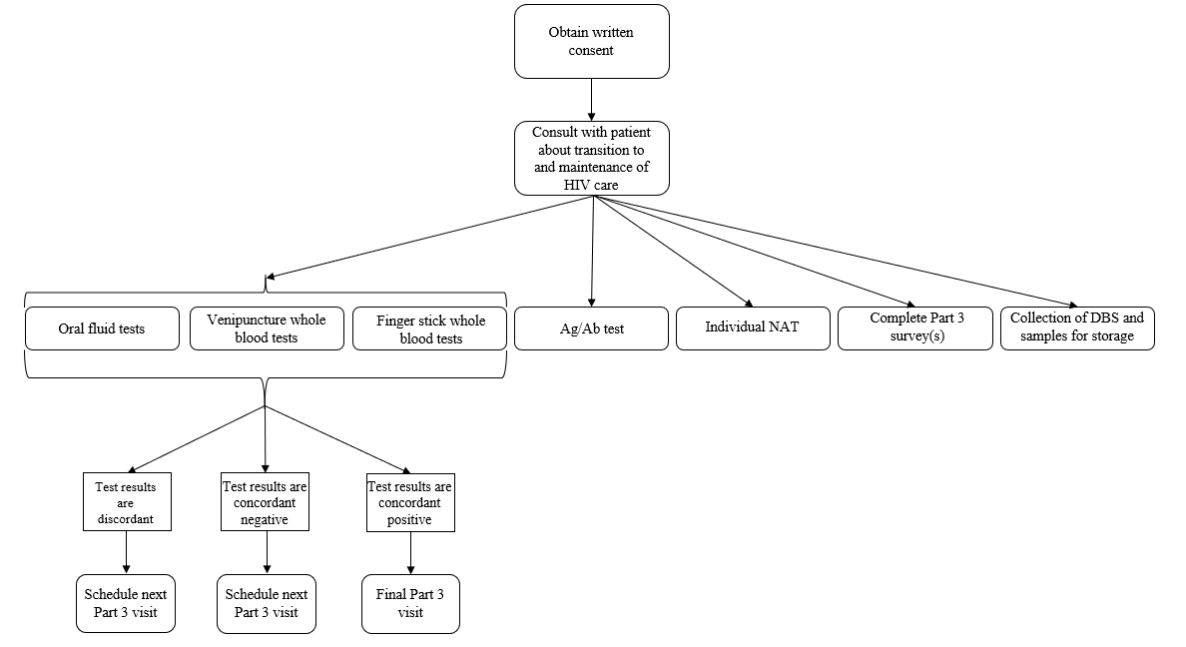
Project DETECT part 3 procedures. Ag/Ab: antigen/antibody; NAT: nucleic acid test; DBS: dries blood spot.

HIV testing procedures for parts 2 and 3 are shown in Table 1 and Figure 2, respectively. When part 2 recruitment began in September 2015, the standard of care HIV testing protocol for the PHSKC STD Clinic was the Genetic Systems HIV-1/2/O IgM-sensitive HIV antibody test (3rd generation; Bio-Rad Laboratories, Inc) followed by pooled HIV nucleic acid testing (NAT) for HIV antibody-negative men who have sex with men (MSM) [11]. On October 12, 2015, the PHSKC laboratory changed to use the HIV-1/HIV-2 Ag/Ab Combo assay (Bio-Rad Laboratories, Inc) for all PHSKC clinic patients, and routine pooling of specimens with negative screening test results was discontinued due to cost concerns. POC testing using the INSTI HIV-1/HIV-2 antibody test (bioLytical Laboratories, Inc) [12] on fingerstick whole blood is offered as part of clinic standard of care selectively to MSM and all persons testing as part of HIV partner services.

Project DETECT study procedures require all participants to undergo POC HIV testing using oral fluid and venipuncture whole blood with results compared to the PHSKC clinic standard of care (Table 1). In addition, all part 3 participants provide fingerstick specimens for POC tests at all visits (Figure 2), and fingerstick specimen testing was added for part 2–group 3 participants after October 19, 2017, following our identification of possible differences between the estimated window periods for fingerstick and venipuncture whole blood specimens that could not be well characterized without a fingerstick specimen collected at the first (part 2) study visit [13]. The devices included in the project remained consistent since the study began in September 2015 except that the Simple Amplification Based Assay (SAMBA, Diagnostics for the Real World), a POC NAT [14,15], was added in June 2018. Specimen collection and testing are performed in accordance with package inserts for each device. For a video demonstrating specimen collection and testing during a mock part 3 visit (including fingersticks) see Multimedia Appendix 2. Part 2 participants with a reactive result on any POC HIV test receive an HIV-1/HIV-2 supplemental test and the clinic standard Ag/Ab laboratory assay and, for part 2–group 1 participants (ie, those with no prior HIV diagnosis), additional blood drawn for a CD4+ T-cell count, HIV-1 RNA level, and connection to PHSKC staff for linkage to HIV care. Laboratory Ag/Ab assays using serum were ordered for all part 2–groups 2 and 3 participants and part 3 participants who were attending their first part 3 visit, had a negative Geenius HIV 1/2 (Bio-Rad Laboratories, Inc) result using either venipuncture or fingerstick whole blood, had no laboratory-based Ag/Ab assay ordered at a previous part 3 visit, or had a previous laboratory-based Ag/Ab result that was nonreactive. Serum specimens are submitted for the Ag/Ab assay under a coded research ID to avoid triggering a public health investigation into persons who are known to be HIV positive.

Since October 12, 2015, when the PHSKC HIV/STD Program discontinued pooled NAT, part 2–group 1 participants who test negative on all POC tests are pooled [11] into a single 10-member pool and then tested using the RealTi*m*e HIV-1 viral load assay (Abbott Laboratories). Plasma is sent for quantitative HIV-1 RNA level for any part 2 participant with any reactive HIV test to confirm HIV infection. Initially, part 3 participants had only one additional HIV-1 RNA test that was performed at the final visit. After the introduction of SAMBA in June 2018, an HIV-1 RNA level has been sent at every part 3 visit. Project DETECT staff provide part 2 participants with all POC test results and, for part 2–group 1 participants, enter these results into the PHSKC electronic medical record.

**Table 1:** Project DETECT part 2 procedures.

Study procedure	Group 1: HIV negative or unknown	Group 2: established HIV infection (>90 days since diagnosis)	Group 3: newly diagnosed HIV positive (≤90 days since diagnosis)
Obtain verbal consent	X	X	
Obtain written consent			X
Release of information to obtain previous HIV results^a^			X
Oral fluid tests^b^	X	X	X
Venipuncture whole blood tests^c^	X	X	X
Fingerstick whole blood tests^d^			X
Geenius HIV 1/2 confirmatory test^e^	X^f^	X	X
Collection of DBS^g^ and samples for storage^h^	X^i^	X	X
Laboratory Ag/Ab^j^ test performed through STD^k^ clinic^l^	X^m^		
Laboratory Ag/Ab test performed through research^l^		X	X
Pooled NAT^n,o^	X^p^		
Individual NAT^q^	X^r^	X	X
Complete part 2 of behavioral survey	X	X	X
Offer and consent, if applicable, part 3 enrollment if POC^s^ tests are discordant	X		X

^a^Release of information is sent to the provider who performed first positive HIV test and/or last negative HIV test if within 365 days.

^b^Oral fluid tests include Dual Path Platform (DPP) HIV 1/2 Assay (Chembio Diagnostics System, Inc) and OraQuick ADVANCE Rapid HIV 1/2 Antibody test (Orasure Technologies, Inc).

^c^Venipuncture whole blood tests include DPP HIV 1/2 Assay, OraQuick ADVANCE Rapid HIV 1/2 Antibody test, INSTI HIV-1/HIV-2 Rapid Antibody Test (bioLytical Laboratories, Inc), Determine HIV 1/2 Ag/Ab Combo (Abbott Laboratories), and SAMBA II HIV-1 Qual test (Diagnostics for the Real World). SAMBA II HIV-1 Qual test was added to the Project DETECT study protocol in June 2018.

^d^Fingerstick whole blood tests include DPP HIV 1/2 Assay, OraQuick ADVANCE Rapid HIV 1/2 Antibody test, INSTI HIV-1/HIV-2 Rapid Antibody Test, Determine HIV 1/2 Ag/Ab Combo, and SAMBA II HIV-1 Qual test. SAMBA II HIV-1 Qual test was added to the Project DETECT study protocol in June 2018.

^e^Geenius HIV 1/2 Supplemental Assay (Bio-Rad Laboratories, Inc).

^f^Geenius HIV 1/2 Supplemental Assay performed as point-of-care test on venipuncture whole blood if at least one point-of-care test is positive.

^g^DBS: dried blood spot.

^h^Includes DPP HIV 1/2 oral fluid swabs, HIV-1 Oral Specimen Collection Device (Orasure Technologies, Inc), and a Whatman 903 Protein Saver Card (dried blood spot; GE Healthcare).

^i^If point-of-care results are discordant, the point-of-care oral fluid and venipuncture whole blood DPP HIV 1/2 Assays are saved and stored.

^j^Ab/Ag: antibody/antigen.

^k^STD: sexually transmitted disease.

^l^GS HIV-1/HIV-2 Combo EIA (Bio-Rad Laboratories, Inc).

^m^If the STD clinic does not order an Ab/Ag test for clinical purposes, the Project DETECT research team will order the test to be performed by PHSKC Public Health Laboratory.

^n^NAT: nucleic acid testing.

^o^10-member pools using RealTi*m*e HIV-1 (Abbott Laboratories).

^p^Pooled NAT is performed only if participant is concordant negative on all point-of-care tests.

^q^RealTi*m*e HIV-1.

^r^Individual NAT is performed if participant has discordant point-of-care test results. Individual NAT has been validated for diagnostic and monitoring purposes.

^s^POC: point-of-care.

#### Specimen Storage and Shipping

At every visit, specimens are collected for processing, storage, and eventual shipping to CDC for creation of a specimen repository to retain samples to evaluate new and yet-to-be-developed HIV tests seeking US Food and Drug Administration (FDA) approval. These specimens include one Dual Path Platform (DPP) oral fluid swab, one OraSure oral fluid collection device, up to ten 1 mL plasma aliquots, and one dried blood spot (DBS) card. In addition, for all part 2 participants with discordant results on POC tests and all part 3 participants, study staff also store the DPP oral fluid swab and DPP whole blood specimens that were used for POC testing.

The repository includes the above specimens from the first 1000 part 2 participants with negative results on all HIV tests and all specimens from participants with at least one reactive HIV test result. These deidentified specimens will be associated with detailed behavioral data (described below) and available for public use in the future.

#### Behavioral Surveys

The PHSKC STD Clinic routinely collects medical and sexual history data from clients seeking clinical services using a computer-assisted self-interview completed at a kiosk in the clinic waiting area. Clients who do not complete the kiosk interview complete a supplemental form during their visit with their clinical provider. As part of the consent process, part 2–group 1 participants agree to have study staff create a link between their clinic and Project DETECT visits to reduce the number of survey questions asked during the study visit and link to HIV test results performed as standard of care at the clinic to the study record.

The three surveys specific to Project DETECT are programmed in Questionnaire Design Studio (Nova Research Company) in which participants are identified only by their study research ID number. Participants complete the part 2 survey, providing electronic affirmation of consent for study participation and specimen storage within the survey as well as demographics; history of HIV testing and recent STDs; symptoms of acute retroviral syndrome [16]; substance use; sexual behaviors, including group sex events [17]; and use of pre-exposure prophylaxis (PrEP), post-exposure prophylaxis (PEP), and ART.

At every part 3 study visit, research staff complete the part 3 Symptom and Care Survey to characterize presence and duration of symptoms associated with acute retroviral syndrome over time as well as the timing of ART initiation; at the initial part 3 visit, the participant completes an affirmation of consent for research participation and specific consent for specimen storage for all specimens to be collected during follow-up. In addition to the Symptom and Care Survey, a part 3 behavioral survey is completed at either the participant’s last study visit, if it occurred prior to visit 9 (approximately 70 days of follow-up), or at visit 9 and at the last study visit when follow-up extends beyond 70 days (Figure 2). At the beginning of the part 3 behavioral survey, research staff complete administrative questions that detail the reason for the final visit and then enter the nicknames of up to three recent male anal sex partners who were reported at the participant’s part 2 visit to collect longitudinal data on partner-specific sexual behaviors. The participant then completes the remainder of the survey. This survey asks similar questions to the part 2 survey and is intended to evaluate changes in behavior following HIV diagnosis.

### Data Collection and Statistical Analysis

Data sources for Project DETECT are shown in Multimedia Appendix 3. For part 1 analyses, Project DETECT staff receive a deidentified dataset from PHSKC staff that includes key sociodemographic variables for all PHSKC STD Clinic patients. The Project DETECT team uses these data to determine the number of clinical patients potentially eligible for Project DETECT, report the participation rate, and describe the characteristics of the overall clinic population.

We present behavioral and demographic data for Project DETECT parts 1 and 2 as descriptive statistics (Table 2). When there is discrepancy, self-reported research data are prioritized over data collected as part of clinical care. When data were not available from the part 2 survey, clinic kiosk data completed by the participants at the clinic visit were used. If neither part 2 data nor clinic kiosk data were available, we used demographics as recorded in the clinic medical record to populate the variable. To define current gender identity, we applied a two-step method involving questions regarding sex assigned at birth and gender identity [18,19]. Participation rates for part 2–group 1 are reported as a proportion of the total STD Clinic population eligible for Project DETECT, although not all enrolled clients may be reflected in the eligible population due to errors or missing data in the clinical database.

**Table 2:** 

Characteristic			Project DETECT^a^ part 2–group 1 participant visits n=1525 n (%)		Project DETECT part 2–group 1–eligible PHSKC^b^ STD^c^ clinic visits^d^ n=14,990 n (%)		Participation percentage	
**Age group in years**								
	18-24			299 (19.6)		2367 (15.8)		12.6
	25-34			667 (43.7)		6588 (44.0)		10.1
	35-44			306 (20.1)		2988 (19.9)		10.2
	45-54			165 (10.8)		2050 (13.7)		8.0
	≥55			88 (5.8)		997 (6.7)		8.8
**Race/ethnicity**								
	Asian			112 (7.3)		1425 (9.5)		7.9
	Black/African American			116 (7.6)		1107 (7.4)		10.5
	Hispanic/Latinx^e^			239 (15.7)		2545 (17.0)		9.4
	Multiracial			56 (3.7)		337 (2.2)		16.6
	Native American			15 (1.0)		107 (0.7)		14.0
	Pacific Islander			11 (0.7)		129 (0.9)		8.5
	White			934 (61.3)		8814 (58.8)		10.6
	Missing/no response			42 (2.8)		526 (3.5)		8.0
**Current gender identity^f^**								
	Cisgender man			1468 (96.3)		14,566 (97.2)		10.1
	Transgender woman			24 (1.6)		130 (0.9)		18.5
	Transgender man			6 (0.4)		56 (0.4)		10.7
	Nonbinary/genderqueer			24 (1.6)		217 (1.4)		11.1
	Other			3 (0.2)		21 (0.1)		14.3
**Sexual orientation**								
	Bisexual			190 (12.5)		1540 (10.3)		12.3
	Gay			1109 (72.7)		11,188 (74.6)		9.9
	Queer			60 (3.9)		585 (3.9)		10.3
	Straight/heterosexual			33 (2.2)		265 (1.8)		12.5
	Other			21 (1.4)		215 (1.4)		9.8
	Missing/no response			112 (7.3)		1197 (8.0)		9.4
**Reason for STD clinic visit (check all that apply)**								
	HIV follow-up visit^g^			59 (3.9)		537 (3.6)		11.0
	Symptoms			361 (23.7)		3907 (26.1)		9.2
	Want to be tested for STD			1005 (65.9)		8445 (56.3)		11.9
	Referred from another clinic or doctor			30 (2.0)		493 (3.3)		6.1
	Treatment or follow-up testing for STD			83 (5.4)		1349 (9.0)		6.2
	Research visit			177 (11.6)		755 (5.0)		23.4
	Contacted by the health department			59 (3.9)		804 (5.4)		7.3
	Want to be tested for HIV			930 (61.0)		6257 (41.7)		14.9
	Other			185 (12.1)		2465 (16.4)		7.5
	Missing/no response			171 (11.2)		1143 (7.6)		15.0
**STD diagnoses during the past year (check all that apply)**								
	Chlamydia			281 (18.4)		3747 (25.0)		7.5
	Gonorrhea			281 (18.4)		3997 (26.7)		7.0
	Syphilis			98 (6.4)		1841 (12.3)		5.3
	Missing/no response			171 (11.2)		1143 (7.6)		15.0
**Substance use during the past year (check all that apply)**								
	**Injection**							
		Heroin		55 (3.6)		193 (1.3)		28.5
		Methamphetamine		106 (7.0)		610 (4.1)		17.4
		Other drugs		37 (2.4)		172 (1.1)		21.5
	**Noninjection**							
		Methamphetamine		180 (11.8)		1199 (8.0)		15.0
	Missing/no response			171 (11.2)		1143 (7.6)		15.0
**Ever tested for HIV?**								
	Yes			1450 (95.1)		13,360 (89.1)		10.9
	No			51 (3.3)		429 (2.9)		11.9
	Missing/no response			24 (1.6)		1201 (8.0)		2.0
**Ever taken PrEP^h^**								
	Yes			441 (28.9)		5323 (35.5)		8.3
	No			1055 (69.2)		6613 (44.1)		16.0
	Missing/no response			29 (1.9)		3054 (20.4)		0.9

^a^DETECT: Diagnostic Evaluation To Expand Critical Testing Technologies.

^b^PHSKC: Public Health–Seattle & King County.

^c^STD: sexually transmitted disease.

^d^There are 27 Project DETECT part 2–group 1 participant visits that were recruited for Project DETECT based on anecdotal evidence of reported sex with men. There is no data evidence of reported sex with men but were included in the Project DETECT part 2–group 1–eligible PHSKC STD Clinic population because they enrolled in Project DETECT.

^e^Persons who identified as Hispanic or Latinx ethnicity were classified as Hispanic/Latinx regardless of race.

^f^Transgender women and transgender men have higher rates of misclassification due to inconsistent reports of sex at birth and current gender identity between the PHSKC STD Clinic kiosk and medical record.

^g“^HIV follow-up visit” was removed as a response option in December 2018.

^h^PrEP: pre-exposure prophylaxis.

## Results

From September 2015 to March 2019, 1331 unique people completed 1819 Project DETECT part 2 research visits. During this period, there were 34,820 visits to the PHSKC STD Clinic by clients not known to be HIV positive, of whom 14,990 (43.05%) were considered to have been eligible for part 2–group 1 enrollment (Table 2). Of the part 2–group 1 eligible visits, 1037 unique people were enrolled and had a total of 1525 part 2–group 1 visits (Table 2). Of the 1037 unique people, 777 participants were seen for a single Project DETECT visit, 151 participants had two visits, and 109 participants had three or more visits. Overall, eligible STD Clinic clients were estimated to participate in approximately 10.17% (1525/14,990) of visits. Participation rates varied by client characteristics: higher rates were seen among younger persons; clients who reported attending the STD Clinic for a research visit (possibly including Project DETECT) or because they wanted to be tested for HIV; clients with no history of PrEP use; and those who reported substance use, specifically injection of heroin, during the past year. Lower rates of participation were seen among persons who had been diagnosed with at least one bacterial STD in the last year, were referred from another provider, were seeking treatment or follow-up testing for an STD, or had been contacted by the health department and asked to come into the STD Clinic.

During the same time period, 198 and 96 participants enrolled in part 2–groups 2 and 3, respectively (Table 3). The majority (241/294, 82.0%) of these participants were recruited from the ACTU or the Madison Clinic, a Ryan White–funded HIV clinic. Characteristics of Project DETECT participants were similar to those of current King County residents living with diagnosed HIV infection [20]. Self-reported STD diagnoses in the previous 3 months ranged between 2.7% (8/294) and 7.8% (23/294) for part 2–groups 2 and 3 participants, with gonorrhea reported most often. More than half (168/294, 57.1%) of the participants reported current ART use; 35.4% (104/294) were ART-naïve at their study visit, the majority of whom were newly diagnosed with HIV (data not shown); 3.7% (11/294) had been ARV-exposed but were not currently on treatment at the time of their study visit; the remaining 3.7% (11/294) had no information on current treatment status.

Twenty-seven persons had discordant HIV test results in part 2 and enrolled in part 3 (Table 4). Participants were followed for a median of 33 days (IQR 10-209 days). Of the 22 participants who were truly HIV positive, 16 (73%) were on treatment at their first part 3 visit and 6 (27%) were ART-naïve. By their last visit, 4 (67%) of the 6 ART-naïve participants had started treatment while 2 (33%) remained ART-naive. Of 27 enrolled participants, 23 (85%) completed the full follow-up period per protocol, including 5 participants with false-positive test results. There were 10 of 27 (37%) participants who tested concordant positive at their final part 3 visit and were considered to be fully seroconverted. There were 8 participants who were discordant through follow-up: one had persistent discordance and 7 had persistent discordance with partial seroreversion, meaning at least one of the POC tests was positive and then became negative at a subsequent visit.

Specimens from Project DETECT are currently maintained in a repository at the CDC and are described by confirmed HIV status after the study visit, HIV test results at the study visit, and self-reported ARV status (Multimedia Appendix 4).

Preliminary data describing HIV test results and partial results from behavioral surveys are presented elsewhere [13,17,21-23]. A description and discussion of one Project DETECT participant with false-positive test results while receiving PrEP has been recently published [24].

**Table 3:** Characteristics of Project DETECT part 2–group 2 and group 3 participants (September 2015 to March 2019) compared with current King County residents living with diagnosed HIV infection as of December 31, 2017.

Characteristics				Project DETECT^a^ group 2 and 3 participants n=294 n (%)		Current King County residents living with diagnosed HIV infection, Dec 2017^b^ n=6907 n (%)
**Project DETECT part 2 participants**						
	Group 2: patients with established HIV infection		198 (67.3)		—	
	Group 3: patients with acute or newly diagnosed HIV infection		96 (32.7)		—	
**Referral site/process**						
	AIDS Clinical Trial Unit/Madison Clinic		241 (82.0)		—	
	PHSKC^c^ STD^d^ clinic		38 (12.9)		—	
	Other site		1 (0.3)		—	
	Missing		14 (4.8)		—	
**Age group in years**						
	18-24		26 (8.8)		122^e^ (1.8)	
	25-34		52 (17.7)		948 (13.7)	
	35-44		47 (16.0)		1445 (20.9)	
	45-54		74 (25.2)		2209 (32.0)	
	≥55		59 (20.1)		2169 (31.4)	
	Missing^f^		36 (12.2)		0 (0)	
**Race/ethnicity**						
	Asian		4 (1.4)		303 (4.4)	
	Black/African American		84 (28.6)		1340 (19.4)	
	Hispanic/Latinx^g^		39 (13.3)		924 (13.4)	
	Multiracial		15 (5.1)		387 (5.6)	
	Native American		10 (3.4)		50 (0.7)	
	Pacific Islander		2 (0.7)		27 (0.4)	
	White		94 (32.0)		3876 (56.1)	
	Missing		46 (15.6)		0 (0)	
**Current gender identity**						
	Cisgender man		232 (78.9)		6004^h^ (86.9)	
	Cisgender woman		43 (14.6)		839^h^ (12.1)	
	Transgender woman		4 (1.4)		59 (0.9)	
	Transgender man		0 (0)		5 (0.1)	
	Nonbinary/genderqueer^i^		4 (1.4)		—	
	Missing		11 (3.7)		0 (0)	
**Gender of sex partners in last year**						
	**Cisgender male participants**					
		Men only	139 (47.3)		—	
		Women only	37 (12.6)		—	
		Men and women	18 (6.1)		—	
		Men and other partners	2 (0.7)		—	
		Men and transgender men	2 (0.7)		—	
		Men, women, and transgender women	2 (0.7)		—	
		Men, women, and transgender men	1 (0.3)		—	
		Women and transgender men	1 (0.3)		—	
		Women and transgender women	1 (0.3)		—	
		Men, women, transgender men, and transgender women	1 (0.3)		—	
	**Cisgender female participants**					
		Men only	29 (9.9)		—	
		Women only	1 (0.3)		—	
		Men and women	1 (0.3)		—	
		Men and other partners	2 (0.7)		—	
	**Transgender female participants**					
		Men only	3 (1.0)		—	
		Men and nonbinary/genderqueer partners	1 (0.3)		—	
	**Nonbinary/genderqueer participants**					
		Men only	1 (0.3)		—	
		Men and women	1 (0.3)		—	
		Men and nonbinary/genderqueer partners	1 (0.3)		—	
		Men, women, transgender men, and other partners	1 (0.3)		—	
		No sex partners	36 (12.2)		—	
		Missing	13 (4.4)		—	
**Sexual orientation**						
	Bisexual		34 (11.6)		—	
	Gay		128 (43.5)		—	
	Queer		4 (1.4)		—	
	Straight/heterosexual		91 (31.0)		—	
	Other		1 (0.3)		—	
	Missing		36 (12.2)		—	
**Self-reported ART** ^j^ **status**						
	ART-naïve		104 (35.4)		—	
	ART-experienced but not currently on ART		11 (3.7)		—	
	Currently on ART		168 (57.1)		—	
	Missing		11 (3.7)		—	
**HIV RNA level at study visit**						
	Undetectable or <40 copies/mL		144 (49.0)		—	
	40-200 copies/mL		14 (4.8)		—	
	201-1000 copies/mL		10 (3.4)		—	
	>1000 copies/mL		119 (40.5)		—	
	Specimen not available^k^		7 (2.4)		—	
**Self-reported STD diagnosis in previous 3 months**						
	Chlamydia		18 (6.1)		—	
	Gonorrhea		23 (7.8)		—	
	Syphilis		22 (7.5)		—	
	Other STD		8 (2.7)		—	
	Missing		15 (5.1)		—	
**Substance use in previous 3 months**						
	**Injection**					
		Heroin	38 (12.9)		—	
		Methamphetamine	69 (23.5)		—	
		Other drugs	6 (2.0)		—	
	**Noninjection**					
		Methamphetamine	80 (27.2)		—	
	Missing		15 (5.1)		—	

^a^DETECT: Diagnostic Evaluation To Expand Critical Testing Technologies.

^b^Data are from the HIV/AIDS Epidemiology Unit, Public Health–Seattle & King County and the Infectious Disease Assessment Unit, Washington State Department of Health. HIV/AIDS Epidemiology Report 2018, Volume 87.

^c^PHSKC: Public Health–Seattle & King County.

^d^STD: sexually transmitted disease.

^e^Includes unspecified number of persons ages 13 to 17 years.

^f^Age of participants is confirmed by medical record in the absence of a part 2 behavioral survey.

^g^Persons who identified as Hispanic or Latinx ethnicity were classified as Hispanic/Latinx regardless of race.

^h^Data do not specify cisgender.

^i^Category not used in PHSKC HIV/AIDS Epidemiology Report 2018.

^j^ART: antiretroviral therapy.

^k^No venipuncture whole blood was drawn at the study visit.

**Table 4:** Project DETECT part 3 enrollment and follow-up (September 2015 to March 2019).

Part 3 follow-up			Project DETECT^a^ part 3 participants n=27 n (%)
Enrolled			27 (100)
**Completed follow-up**			23 (85.2)
	False positive^b^		5 (18.5)
	Concordant positive results with full seroconversion		10 (37.0)
	**Discordant through follow-up**		8 (29.6)
		Persistent discordance without seroreversion	1 (3.7)
		Persistent discordance with partial seroreversion^b^	7 (25.9)
Lost to follow-up			4 (14.8)

^a^DETECT: Diagnostic Evaluation To Expand Critical Testing Technologies.

^b^A false positive is confirmed by a negative viral load result (target not detected) by the RealTi*m*e HIV-1 viral load assay (Abbott Laboratories).

^c^Seroreversion is defined as having at least one point-of-care test result that is positive or reactive followed by a negative or nonreactive test result at a subsequent visit.

## Discussion

### Principal Findings

Project DETECT is a study designed to evaluate the performance of POC HIV tests with oral fluids and venipuncture and fingerstick whole blood specimens and establish a specimen and data repository. This manuscript describes the current protocol and initial populations of persons recruited for study participation.

In the first 2 years of study enrollment, Project DETECT enrolled 1525 visits with persons who were HIV negative or unknown status. While these individuals represent a small proportion (10%) of the 14,990 eligible visits to the PHSKC STD Clinic, willingness to participate was high among people who were approached by study staff (data not shown). Clients seeking HIV testing may have been more motivated to participate, and some clients may have been aware of the study from prior participation or word of mouth and attended the clinic specifically to participate in the study. Although a recent bacterial STD is a strong predictor of HIV acquisition, clients diagnosed with at least one bacterial STD were underrepresented in the study population [25]. There also may have been time, structural, or other barriers to enrolling clients in a research project when they needed to prioritize seeing a clinician for STD care.

During this same time, Project DETECT enrolled 294 persons into part 2–groups 2 and 3 who had established or newly diagnosed HIV infection. A panel of specimens is available from this group, who include HIV-positive persons who are ARV-naïve, ARV-experienced but not currently receiving ART, and ARV-treated. The number of HIV-infected participants and available specimens will increase as enrollment continues.

Participants also continue to enroll into longitudinal follow-up in part 3 when they have discordant HIV results in part 2, providing critical data on the window periods of different HIV tests and specimen types when performed in real time. Although we and others have evaluated POC tests in the past, either in cross-sectional studies [10,26] or on frozen plasma specimens [27-30], to our knowledge this is the first project to evaluate these tests longitudinally and in real time using the unprocessed specimens typical of POC testing. These data are necessary for CDC to provide guidance on the use of POC HIV tests and recommendations on the period for retesting persons at risk for HIV infection based on the test and specimen type.

Project DETECT is also the first research study in the United States to evaluate SAMBA, a POC NAT [14,15], for use in screening high-risk persons for acute HIV infection (including persons on PrEP) as well as its ability to monitor HIV-positive persons on ART for virologic failure. As new HIV test technologies and diagnostic approaches performed on unprocessed whole blood or oral fluid continue to reach the US market, the protocol described here can serve as a model for evaluating their performance.

### Limitations

Project DETECT does have limitations that could impact the reproducibility or generalizability of some of our future findings. The majority of the HIV-negative study population consists primarily of cisgender MSM because of the epidemiology of HIV infection in Seattle, Washington [20], although there is no evidence that HIV test sensitivity varies by gender. Similarly, most HIV-positive participants are presumed to have been infected with subtype B virus, when it is possible they have another subtype. Finally, the high level of PrEP uptake among HIV-negative participants and ART among people with HIV in Seattle [31-33] may impact our estimates of the sensitivity, specificity, and window periods of the different HIV tests. However, these results provide critical information on test performance in the context of PrEP as communities across the United States and elsewhere continue to bring PrEP to scale. In particular, the use of SAMBA in Project DETECT will provide much needed information on the utility of screening for cell-associated DNA in addition to plasma RNA in populations receiving PrEP.

### Next Steps

Additional analyses based on the Project DETECT protocol described here are in progress. Future reports will include descriptions of HIV test sensitivity, specificity, and window periods; an in-depth evaluation of SAMBA; and analyses of behavioral data to evaluate risks associated with participation in group sex events. Furthermore, the specimen repository and paired behavioral data will be used and disseminated by CDC to evaluate new HIV tests as they become available. The Project DETECT specimen repository now includes oral fluids, plasma, whole blood, and DBS from the first 1000 part 2 study visits in which participants tested HIV negative on all tests, from 390 part 2 visits in which participants had at least one HIV-positive test result, and from all 206 part 3 visits.

In July 2018, the FDA’s Blood Products Advisory Committee met to discuss the potential reclassification of HIV POC and laboratory-based serological and NAT diagnostic devices from Class III (high-risk devices) to Class II (moderate-risk devices) [34]. If enacted, this reclassification will reduce regulatory burden and facilitate timelier access to HIV testing devices for clinicians and patients. CDC will continue to use data from Project DETECT or similar protocols to evaluate and report on the performance of POC HIV tests, including those that become available in the US market following reclassification.

## Appendix

Multimedia Appendix 1 Project DETECT part 3 visit schedule.

Multimedia Appendix 2 Project DETECT specimen collection and HIV testing.

Multimedia Appendix 3 Characteristics of Project DETECT part 2 data.

Multimedia Appendix 4 Laboratory specimens from Project DETECT participants stored in the US Centers for Disease Control and Prevention specimen repository (September 2015 to September 2019).

## Abbreviations

ACTU: AIDS Clinical Trials Unit
Ag/Ab: antigen/antibody
ART: antiretroviral therapy
ARV: antiretroviral
CDC: US Centers for Disease Control and Prevention
DBS: dried blood spot
DDP: dual path platform
FDA: US Food and Drug Administration
IQR: interquartile range
MSM: men who have sex with men
NAT: nucleic acid test
PEP: postexposure prophylaxis
PHSKC: Public Health–Seattle & King County
POC: point-of-care
PrEP: pre-exposure prophylaxis
Project DETECT: Diagnostic Evaluation To Expand Critical Testing Technologies
SAMBA: Simple Amplification Based Assay
STD: sexually transmitted disease
UW: University of Washington
