# Supplementary material for: Prospective Evaluation of HIV Testing Technologies in a Clinical Setting: Protocol for Project DETECT
Source: JMIR Res Protoc. 2020 Jan 27;9(1):e16332. doi: 10.2196/16332 (PMC7011122; doi:10.2196/16332)
Supplement: Multimedia Appendix 1 [file resprot_v9i1e16332_app1.docx]

Multimedia Appendix 1. Project DETECT part 3 visit schedule.

| **Visit Number** | **Visit Window Opens** | **Visit Target** | **Visit Window Closes** |
| --- | --- | --- | --- |
| Part 2 Visit | N/A | Day 0 | N/A |
| Part 3 Visit #1 | Day 1 | Day 3 | Day 19 |
| Visit #2 | Day 4 | Day 7 | Day 20 |
| Visit #3 | Day 8 | Day 10 | Day 21 |
| Visit #4 | Day 11 | Day 14 | Day 28 |
| Visit #5 | Day 15 | Day 21 | Day 55 |
| Visit #6 | Day 22 | Day 28 | Day 56 |
| Visit #7 | Day 29 | Day 42 | Day 82 |
| Visit #8 | Day 43 | Day 56 | Day 83 |
| Visit #9 | Day 57 | Day 70 | Day 84 |
| Visit #10 | Day 78 | Day 90 | Day 106 |
| Visit #11 | Day 107 | Day 120 | Day 135 |
| Visit #12 | Day 137 | Day 150 | Day 165 |
| Visit #13 | Day 168 | Day 180 | Day 196 |
| Visit #14 | Day 198 | Day 210 | Day 226 |
| Visit #15 | Day 229 | Day 240 | Day 257 |
| Visit #16 | Day 259 | Day 270 | Day 288 |
| Visit #17 | Day 289 | Day 300 | Day 318 |
| Visit #18 | Day 320 | Day 330 | Day 349 |
| Visit #19 | Day 351 | Day 365 | Day 365 |
