# Supplementary material for: Prospective Evaluation of HIV Testing Technologies in a Clinical Setting: Protocol for Project DETECT
Source: JMIR Res Protoc. 2020 Jan 27;9(1):e16332. doi: 10.2196/16332 (PMC7011122; doi:10.2196/16332)
Supplement: Multimedia Appendix 3 [file resprot_v9i1e16332_app3.docx]

Multimedia Appendix 3. Characteristics of Project DETECT part 2 data.

| **Type of Data** | **Source** |
| --- | --- |
| Group 1 | |
| Demographics | Self-reported in the PHSKC STD Clinic kiosk survey, electronic medical record, or Part 2 behavioral survey |
| Point-of-Care HIV Test Results | PHSKC STD Clinic electronic medical record |
| Laboratory HIV Test Results | PHSKC STD Clinic electronic medical record ^a^ and Project DETECT test result database ^b^ |
| Behavioral Survey | Self-reported in the Part 2 behavioral survey |
| Previous HIV Testing History | Self-reported in the Part 2 behavioral survey |
| Groups 2 and 3 | |
| Demographics | Participant-completed Part 2 behavioral survey |
| Point-of-Care HIV Test Results | Project DETECT test result database ^c^ |
| Laboratory HIV Test Results | Project DETECT test result database ^c^ |
| Behavioral Survey | Participant-completed Part 2 behavioral survey |
| Previous HIV Testing History | Self-reported in the Part 2 behavioral survey or medical records from the previous year ^d^ |
