# Supplementary material for: Prospective Evaluation of HIV Testing Technologies in a Clinical Setting: Protocol for Project DETECT
Source: JMIR Res Protoc. 2020 Jan 27;9(1):e16332. doi: 10.2196/16332 (PMC7011122; doi:10.2196/16332)
Supplement: Multimedia Appendix 4 [file resprot_v9i1e16332_app4.docx]

Multimedia Appendix 4. Laboratory specimens from Project DETECT participants stored in the CDC specimen repository, September 2015-September 2019

| **Specimen type** | **Confirmed HIV-negative** ^a^ | | | | **Confirmed HIV positive** ^a^ | | | | | | **Total number of specimens** |
| --- | --- | --- | --- | --- | --- | --- | --- | --- | --- | --- | --- |
|  | **Concordant negative study test results** | | **Confirmed false positive study test results ^b^** | | **Concordant positive study test results** | | | **Discordant study test results** | | |  |
|  | **On PrEP ^c^** | **Not on PrEP ^c^** | **On PrEP ^c^** | **Not on PrEP ^c^** | **ART-Naïve ^d^** | **Currently on ART ^d^** | **Not currently on ART ^d^** | **ART-Naïve ^d^** | **Currently on ART ^d^** | **Not currently on ART ^d^** |  |
| Oral fluid |  | | | | | | | | | | |
| Orasure ^e^ | 142 | 877 | 13 | 21 | 109 | 223 | 18 | 24 | 177 | 1 | 1605 |
| DPP OMT ^f^ | 144 | 888 | 25 | 37 | 121 | 235 | 18 | 45 | 351 | 2 | 1866 |
| Whole blood |  | | | | | | | | | | |
| DPP WB ^g^ | 2 | 9 | 12 | 16 | 11 | 11 | 0 | 21 | 171 | 1 | 254 |
| DPP FS ^h^ | 2 | 8 | 9 | 10 | 0 | 10 | 0 | 12 | 161 | 1 | 213 |
| Plasma ^i^ | 1414 | 8654 | 126 | 200 | 964 | 1935 | 159 | 226 | 1572 | 8 | 15258 |
| DBS ^j^ | 142 | 876 | 13 | 21 | 108 | 219 | 18 | 24 | 177 | 1 | 1599 |

PrEP: pre-exposure prophylaxis; ART: antiretroviral therapy.

^a^ HIV status is confirmed using the CDC testing algorithm.

^b^ Of the 34 visits with false positive study test results, 26 visits had false positive whole blood results, 2 had false positive oral fluid results, and 6 had false positive laboratory-based Ag/Ab test result.

^c^ Current PrEP use at time of study visit is self-reported. Participants with missing survey information were classified as “Not on PrEP” in Table 5.

^d^ Current ART use at time of study visit is self-reported in either the participant’s behavioral survey, or on the participant study record if a survey was missing from the study visit.

^e^ HIV-1 Oral Specimen Collection Device (OraSure Technologies, Inc., Bethlehem, PA, US) containing up to 1.25mL of oral fluid.

^f^ Up to two DPP HIV 1/2 oral fluid specimens (Chembio Diagnostics System, Inc., Medford, NY, US) containing up to 1.25mL of oral fluid each.

^g^ DPP HIV 1/2 venipuncture whole blood specimen (Chembio Diagnostics System, Inc., Medford, NY, US) containing up to 1.25mL of sample.

^h^ DPP HIV 1/2 finger stick whole blood specimen (Chembio Diagnostics System, Inc., Medford, NY, US) containing up to 1.25mL of sample.

^i^ Up to ten plasma aliquots per participant, each containing up to 1mL of plasma.

^j^ Whatman 903 Protein Saver Card (Dried Blood Spot) (GE Healthcare, Chicago, IL, US).
